# White matter microstructural abnormalities and default network degeneration are associated with early memory deficit in Alzheimer’s disease continuum

**DOI:** 10.1038/s41598-019-41363-2

**Published:** 2019-03-18

**Authors:** Fang Ji, Ofer Pasternak, Kwun Kei Ng, Joanna Su Xian Chong, Siwei Liu, Liwen Zhang, Hee Youn Shim, Yng Miin Loke, Boon Yeow Tan, Narayanaswamy Venketasubramanian, Christopher Li-Hsian Chen, Juan Helen Zhou

**Affiliations:** 1 Center for Cognitive Neuroscience, Neuroscience and Behavioural Disorders Program, Duke-National University of Singapore Medical School, Singapore, Singapore; 2 Departments of Psychiatry and Radiology, Brigham and Women’s Hospital, Harvard Medical School, Boston, USA; 3 St Luke’s Hospital, Singapore, Singapore; 4 Raffles Neuroscience Centre, Raffles Hospital, Singapore, Singapore; 5 Department of Pharmacology, National University Health System, Clinical Research Centre, Singapore, Singapore; 6 Memory, Aging & Cognition Centre, National University Health System, National University of Singapore, Singapore, Singapore; 7 Clinical Imaging Research Centre, the Agency for Science, Technology and Research, Singapore, Singapore

## Abstract

Instead of assuming a constant relationship between brain abnormalities and memory impairment, we aimed to examine the stage-dependent contributions of multimodal brain structural and functional deterioration to memory impairment in the Alzheimer’s disease (AD) continuum. We assessed grey matter volume, white matter (WM) microstructural measures (free-water (FW) and FW-corrected fractional anisotropy), and functional connectivity of the default mode network (DMN) in 54 amnestic mild cognitive impairment (aMCI) and 46 AD. We employed a novel sparse varying coefficient model to investigate how the associations between abnormal brain measures and memory impairment varied throughout disease continuum. We found lower functional connectivity in the DMN was related to worse memory across AD continuum. Higher widespread white matter FW and lower fractional anisotropy in the fornix showed a stronger association with memory impairment in the early aMCI stage; such WM-memory associations then decreased with increased dementia severity. Notably, the effect of the DMN atrophy occurred in early aMCI stage, while the effect of the medial temporal atrophy occurred in the AD stage. Our study provided evidence to support the hypothetical progression models underlying memory dysfunction in AD cascade and underscored the importance of FW increases and DMN degeneration in early stage of memory deficit.

## Introduction

Alzheimer’s disease (AD) is a gradual progressive neurodegenerative disorder in which memory deficit is typically the most salient cognitive symptom^1^. Patients with amnestic mild cognitive impairment (aMCI) are at higher risk of developing AD, where aMCI is frequently considered as early stage of AD^2,3^. Converging evidence suggests that both AD and aMCI are associated with large-scale functional network dysconnectivity, especially in the default mode network (DMN), which consists of the posterior cingulate cortex (PCC), precuneus, medial prefrontal cortex (mPFC), and bilateral angular gyrus^4^. DMN dysconnectivity is often associated with worsened memory^4,5^. In parallel, grey matter volume (GMV) loss in the medial temporal lobe (MTL) and DMN regions^6–8^, are typically related to memory decline in AD patients^9,10^. Moreover, diffusion tensor imaging (DTI) studies have revealed that compromised white matter (WM) microstructures, particularly in the corpus callosum, cingulum, and fornix^11^, are associated with memory deficit in AD^12,13^. Recently, free-water (FW) imaging using diffusion MRI data was proposed to address the partial volume effect problem^14^. As a result, FW increases have been associated with extracellular processes such as inflammation and small vascular damage in neurodegenerative diseases^15^. On the other hand, the FW-corrected DTI metrics represent microstructural tissue changes such as degeneration and myelin sheath alterations^16^. However, one critical gap is whether and how these brain structural and functional degenerative processes differ in the temporal sequence of their influence on memory performance in the AD continuum.

The spectrum of AD spans from clinically asymptomatic to severely impaired^17^. Based on the hypothetical AD cascade model^11,18–21^, the influences of abnormal brain imaging measures on memory in AD would be more appropriately considered as a multi-facet process moving along a seamless continuum rather than as discrete clinical stages^1^. Recent evidence suggests that pathophysiological abnormalities of AD precede overt memory decline and progress in a non-linear manner^18,22,23^. For example, atrophy rates of MTL and DMN regions are not uniform across disease stages and they exhibit differential trajectories^24–26^. The vascular damage and neuroinflammatory-related brain changes also vary with AD continuum^11,27,28^. However, previous studies have mostly associated abnormal brain measures with memory decline in AD patients using linear regression models^12,29^. These models assumed a constant linear relationship between brain measures and cognition over stages, which ignored the possibility of varying brain-cognition relationship across the disease spectrum^30^. Taken together, we speculate that the influence of brain abnormities on memory varies according to disease stage. However, the significance of these dynamic associations and their potential role in AD continuum have not been characterized.

To address this gap, we examined the stage-dependent associations between multimodal brain measures and memory decline in AD continuum using a novel sparse varying coefficient (SVC) model^31^. SVC model allows us to use one model to simultaneously compare the trajectories from multiple brain measures^32^. Furthermore, unlike conventional linear models in previous studies^4,11,18^, SVC model does not assume a constant linear association between brain measures and memory performance across stages; instead, it allows the association to vary non-linearly with dementia severity. Specifically, based on prior evidence that WM microstructural abnormalities and functional network degeneration might occur earlier than the MTL atrophy in AD^6,11,19^, we hypothesized that the influence of WM microstructural abnormalities and DMN functional dysconnectivity on memory impairment would take place in the aMCI stage, while the influence of MTL atrophy would be more prominent later.

## Results

### Specific brain structural and functional abnormalities are associated with memory deficit

**Figure 1:**
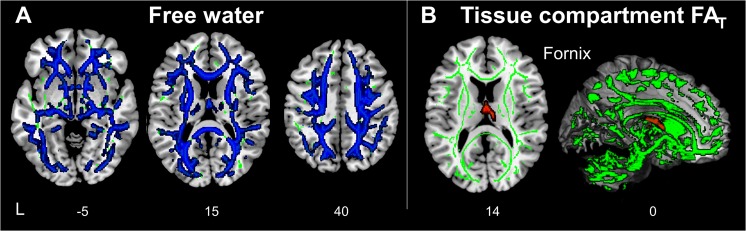
Free-water (FW) increases and tissue compartment fractional anisotropy (FA_T_) deterioration correlated with verbal memory deficit. (**A**) Whole-brain voxel-wise linear regression analysis indicated that higher FW values in widespread brain regions were associated with poorer memory. (**B**) Lower FA_T_ in the body of the fornix was associated with worse memory. The WM skeleton is highlighted in green. All the results are threshold-free cluster enhancement and family-wise error-corrected at p < 0.05.

To determine regions-of-interests for SVC modelling, we performed several whole-brain voxel-wise analyses on the associations between brain abnormalities and memory deficit in patients. The whole-brain voxel-wise analysis on the FW-corrected diffusion MRI metrics showed that lower memory scores in aMCI and AD patients were associated with higher FW in most WM regions. (Fig. 1A, Supplementary Table 1). In contrast, lower memory score was associated with lower fractional anisotropy (FA_T_) in the body of the fornix only (Fig. 1B, Supplementary Table 1).

**Figure 2:**
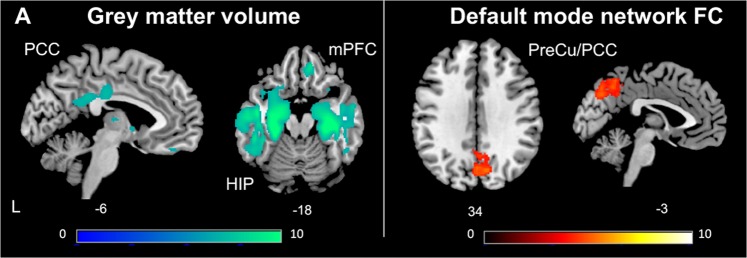
Grey matter volume (GMV) loss and default mode network (DMN) functional connectivity (FC) disruption correlated with memory deficit. (**A**) Whole-brain voxel-wise linear regression analysis indicated that more grey matter atrophy in the hippocampal/parahippocampal (HIP) regions, posterior cingulate cortex (PCC), and medial prefrontal cortex (mPFC) was associated with worse memory (p < 0.05, family-wise error-corrected). (**B**) Lower FC of the DMN in the precuneus (PreCu)/PCC regions was associated with worse memory (height threshold of p < 0.01 and a cluster threshold of p < 0.05, gaussian random field-corrected).

The voxel-wise analysis on grey matter volume revealed that lower GMV in the bilateral MTL (particularly in the HIP), PCC, and mPFC were associated with lower memory scores across all patients (Fig. 2A, Supplementary Table 2).

Finally, the voxel-wise analysis on the DMN FC revealed that lower memory score was associated with lower FC in the precuneus and part of PCC regions across all patients (Fig. 2B, Supplementary Table 3).

These findings remained significant after controlling for years of education. Further details are provided in Supplemental Data (Supplementary Fig. 5, Supplementary Results).

In addition, we found greater brain abnormities (FW, FA_T_, GMV and FC) in AD patients compared with aMCI patients as expected (Supplementary Fig. 2), which included those memory-related brain measures. Further details for group difference results among HC, aMCI and AD are provided in Supplemental Data (Supplementary Results).

### Differential stage-dependent associations of multimodal brain abnormalities with memory performance

To investigate the severity-dependent (CDR- sum of boxes (CDR-SB) to denote dementia severity) contributions of both brain functional and structural measurements simultaneously, we built an SVC model with memory as the dependent variable and FW, FA_T_ in the fornix, GMV-mPFC, GMV-PCC, GMV-HIP, and FC-DMN derived from the significant regions of voxel-wise analysis as predictors.

**Figure 3:**
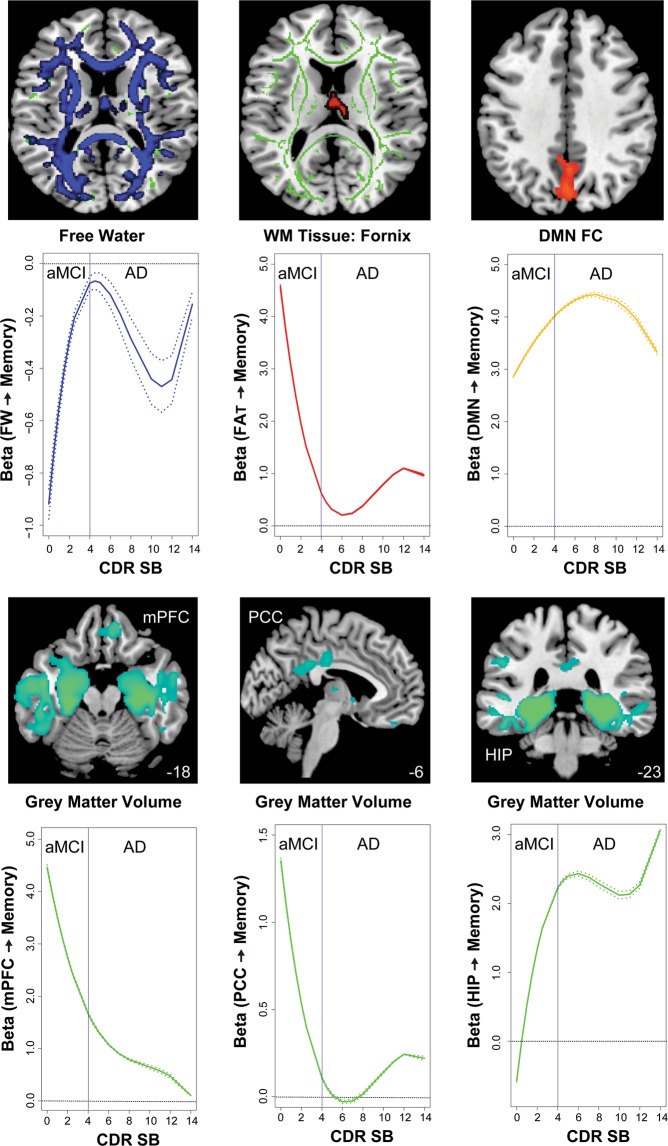
Severity-dependent associations of free water (FW), tissue compartment fractional anisotropy (FA_T_), grey matter volume (GMV), and functional connectivity (FC) with memory performance derived from a sparse varying coefficient model. Solid curves represent the mean associations (Beta coefficients) of brain measurements, with memory as a function of dementia severity (represented by the Clinical Dementia Rating Scale, sum-of-boxes (CDR-SB)) estimated from 100 replicates. The dashed curves represent the point-wise 2* standard errors of the solid curves estimated from 100 replicates. The horizontal dashed black lines represent Beta = 0. Abbreviations: HIP: hippocampus, PCC: posterior cingulate cortex, mPFC: medial prefrontal cortex.

We found these brain measures exhibited differential severity-dependent associations with memory (Fig. 3). For DTI, FW had the greatest influence on memory deficit in the early aMCI phase where higher FW was associated with lower memory score (peak beta = −0.9). However, this influence gradually decreased in late aMCI and AD stage (i.e., less negative betas approaching zero). Similarly, the association of FA_T_ in the fornix with memory score was the greatest in early aMCI stage (peak beta = 4.5), where higher FA_T_ was associated with better memory score. However, this association quickly diminished in the AD stage (i.e., smaller positive betas approaching zero).

For GMV, both PCC and mPFC had the strongest associations with memory in the early aMCI stage, where larger volume was associated with better memory score (mPFC peak beta = 4.5; PCC peak beta = 1.4). Similar to FA_T_, this relationship gradually diminished in the AD stage (i.e., smaller positive betas). In contrast, the relationship between hippocampus (and MTL) and memory were more evident in the late aMCI stage and peaked at the early AD phase (beta = 2.4) where larger volume was associated with better memory (i.e., greater positive betas). The association between FC-DMN and memory was evident throughout the disease continuum. Higher FC was associated with higher memory score regardless of severity (i.e., comparable positive betas).

We also evaluated the specificity of SVC model following our previous approach^32^. We randomly permuted the memory scores 100 times across the subjects and repeated SVC modelling 100 times on each of the 100 permuted data sets (dependent variable: memory z-scores; 10 predictors: brain measures [FW, FA_T_, FC-DMN, GMV-PCC, GMV-mPFC, GMV-HIP] together with nuisance variables [age, gender, handedness and ethnicity]). In 52 out of the 100 permuted data sets, no variable was selected by all 100 repetitions. For each of the remaining 48 permutated datasets, the SVC model selected one variable from 10 predictors as the key predictor of verbal memory scores based on 100 repetitions. However, the frequency distribution of variable selection across these 48 data sets was random. None of the predictors was selected for all 100 repetitions (Supplementary Fig. 3). Overall, the selected variables using our original data set did not favour other variables in the null distribution. This indicates the high specificity of SVC models built on the original dataset.

Lastly, when the years of education was added into the SVC modelling as a covariate, the estimated severity-dependent relationships of all brain regions with memory remained similar as the SVC model without education (Supplementary Results and Supplementary Fig. 6).

## Discussion

The present study demonstrated differential stage-dependent associations between brain structural/functional abnormalities and memory impairment over the course of AD progression using SVC model. Our findings support the hypothetical model of sequential but temporally overlapping multimodal brain abnormality cascades in AD^11,18–21^. A key advantage of SVC model^32^ is the use of one multivariant model to compare the stage-varying influences of FW increases, fornix degeneration, GM atrophy of MTL and DMN hubs, and DMN dysfunction on memory deficit as AD progresses. This model does not require the assumption of constant brain-cognition relationships over disease progression; instead, it captures the nonlinear trajectories of these relationships. Specifically, lower FA_T_ and higher FW had stronger associations with memory deficit in patients at early aMCI stage (in contrast to AD stage). Similarly, atrophy in the DMN (mPFC and PCC) was more strongly associated with memory deficit in patients with aMCI. In contrast, GMV loss in the MTL was more strongly associated with poor memory in the AD phase. Compared to the stage dependence in the structural measures, an association between DMN functional disconnections and memory impairment persisted throughout AD progression. Our findings provide new insight into the multifaceted neurobiological mechanisms underlying memory dysfunction along the AD continuum and highlight the potential importance of WM microstructural abnormalities and DMN degeneration in early cognitive deterioration. Multimodal neuroimaging assays could be further developed to track the efficacy of early cognitive intervention strategies.

Consistent with the hypothesis that early WM dysfunction appears in the early stage of AD^11^, our results demonstrated that microstructural WM measures were associated with memory performance in the aMCI phase. Importantly, by applying the free-water imaging method, we further demonstrated that two different WM pathophysiological measures were associated with memory deficit in the aMCI stage: higher global FW and focal tissue damage in the body of the fornix. Previous studies demonstrated widespread FW increases in AD and aMCI subjects as compared with HC subjects^15,33^. This water content increase may be due to microvascular degeneration^11,34^ and neuroinflammation-related modulation of the blood-brain barrier permeability^35^ in the widespread WM tissues of AD patients. However, the functional significance of such an increase in FW is not well understood. In this study, we showed widespread increase in FW was associated with memory deficit, particularly in the aMCI stage. This suggests that widespread small vascular degeneration and/or chronic neuroinflammation might play important roles in memory deficit during the early stage of AD^36^. Additionally, we observed a slight ‘bump’ of the FW-memory association in the clinical phase (relatively more negative betas at the AD stage in Fig. 3). These two phases of stronger FW-memory association during disease progression are in line with outcomes from a recent study where both early and late peaks of microglial activation (which triggers inflammation) were involved in prodromal and clinical stages of AD^27^, respectively. Therefore, our results might suggest the potential role of FW increases in memory deficit in the early aMCI stage.

Another important finding in our study is that the FA_T_ in the body of the fornix is associated with memory deficit. Our SVC model showed that this association peaked at aMCI and then decreased during the AD stage. The fornix is a predominant tract connecting the hippocampus to the septal nuclei and the mammillary bodies in the hypothalamus. It is particularly susceptible to pathological assaults and shows early changes in AD^37^. Moreover, the fornix microstructure has been used to classify AD diagnosis and assess cognitive changes and response to therapy in both human^13^ and animal models^38^. Recent studies have demonstrated that fornix microstructure accounts for both age-related and age-independent variations in free recall test^39^. A prior longitudinal study also indicated that FA in the fornix could predict memory decline and progression to AD in MCI patients^12^. Of note, this focal fornix tissue damage had greater association (in term of beta) with memory deficit than the global FW increase, which suggests that memory-related WM tract deterioration may play a more dominant role than the widespread ‘background’ vascular/inflammatory damage in memory performance decline. Therefore, our SVC results further bolstered the plausibility that the fornix may be one of the earliest damaged regions that potentially contribute to worse memory outcome in AD.

In contrast to the stronger influence of hippocampal atrophy in AD stage, we found atrophy in the DMN hubs (mPFC and PCC) to be more strongly associated with poorer memory performance in the aMCI phase. Past studies have reported both MTL atrophy and DMN damage occurs at the early stage of AD^6,40^. However, the stage-dependant contribution of these GM regions to memory deficits remains unknown. Using the multivariant SVC model that combined mPFC, PCC and MTL regions, our findings provide evidence that DMN atrophy may have greater influence to memory decline at the aMCI stage, while MTL atrophy has greater contribution at AD stage. Furthermore, studies have demonstrated that GM atrophy mediates the effects of amyloid and Tau on memory^41,42^. Our results on the differential stage-dependent atrophy-memory association are consistent with the pathophysiological mechanisms of AD progression: neuronal degeneration in the DMN related to early amyloid burden and hypometabolism and medial temporal atrophy related to later Tau pathology in the clinical stage of AD^7,18,43^. Both hippocampus and DMN hubs functionally support complementary functions in episodic memory. The hippocampus organizes memories in the context in which they were experienced (a defining feature of episodic memory), whereas the DMN hubs control the retrieval of memories by suppressing competing memories and are responsible for flexibly switching between memory ‘tracks’ according to contextual rules^9,44^. Indeed, interference suppression and retrieval processes have been compromised in healthy elderly and patients with aMCI^45,46^, consistent with the observation of an early stronger GMV-memory association in the DMN than in the MTL. Additionally, we observed that mPFC had slightly higher association (in term of beta) with memory than the PCC at the early aMCI stage, which was consistent with previous literature that prefrontal cortex plays an essential role in the memory processing pathway^9^.

In contrast to the structural measures, the FC of the DMN hubs showed positive associations with memory performance across both prodromal and clinical AD stages. These findings are consistent with prior studies^5,19^. Synaptic dysfunction and grey and white matter deteriorations could impact the functional organization of the DMN and lead to memory deficit^7,10,47^. As a result, the association between PCC-based DMN FC and memory remained relatively stable across disease progression.

Overall, our results suggest a possible mechanism of memory deficit in AD. During the early stage of AD, the structure and function of the DMN hubs (particularly PCC and mPFC) may be targeted due to selective vulnerability^48^ and/or early amyloid burden^43^, accompanied by the associated WM deterioration, disconnection with hippocampus, and widespread WM inflammation and vascular damage^15,35,40^. Taken together, these factors may impair memory performance. As AD progresses, the impacts of WM damages to memory would be greatly reduced due to possible ceiling effects^11^. Along with this process, MTL atrophy and more severe functional network breakdown become the dominant factors contributing to further memory impairments^6,18^, supporting the hypothetical AD cascade model^11,18,19^. Therefore, our results implied that extracellular FW increases and DMN degeneration may be the potential targets for early intervention strategies to slow down memory decline in AD, while MTL atrophy in late AD may be used as an imaging marker to monitor progression of memory deficit^18^.

Although we have demonstrated the significance of stage-dependent contributions of multimodal brain structural and functional deterioration to memory impairment in AD progression, our study has limitations. One limitation is that the associations between brain function/structure and memory derived from the cross-sectional dataset may be confounded by inter-subject anatomical variability and not fully reflect within-subject longitudinal stage-dependent brain-cognition associations. However, our findings are consistent with the AD cascade hypothesis and can serve as a working model for future longitudinal studies. Secondly, no amyloid PET imaging or cerebrospinal fluid markers were available for this cohort. Therefore, we could not rule out the possibility of other pathologies besides AD in patients with aMCI and AD. Thirdly, although we used global signal regression to remove physiological noise, residuals of physiological signals could still remain^49,50^. Advanced methods such as RETROICOR^51^ making use of concurrent physiological recordings are needed in the future to mitigate the influence of physiological noise. Fourthly, there was a relatively limited sample size of participants in those bins with severe dementia symptom (CDR-SB > 10), leading to non-uniform CDR-SB distribution (Supplementary Fig. 4), which might affect the estimation accuracy in the SVC modelling at the end of the dementia spectrum. Future studies on larger sample with longitudinal follow-ups would help characterize finer severity-dependent brain-cognition trajectories. Furthermore, the initial screening step of linear regression might miss some brain regions whose structural or functional properties influence memory in a non-linear manner, which require complex statistical modelling to infer nonlinear stage-dependent brain-behaviour relationship^28^. Lastly, compared to the current single shell diffusion MRI data, advanced FW correction based on multi-shell data would further improve the accuracy of FW separation^16^.

## Conclusion

Based on the sequential but temporally overlapping patterns of brain-memory associations, our study supports the hypothetical progression models of multimodality brain integrity related to memory dysfunction in the AD continuum. Furthermore, our results underscore the importance of WM microstructure, extracellular water, and DMN degeneration in the early stage of the disease, which may guide treatment options to slow down cognitive decline.

## Methods

### Ethics approval and consent to participate

This study was conducted in accordance with the Declaration of Helsinki, and written informed consent was obtained from each participant. Ethical approval was provided by the National Healthcare Group Domain-Specific Review Board, Singapore.

### Participants

All patients were recruited from the National University Hospital of Singapore and St. Luke’s Hospital in Singapore^15^. Trained psychologists assessed each participant with a comprehensive clinical and neuropsychological evaluation including the Clinical Dementia Rating Scale (CDR), the Mini-Mental State Examination (MMSE), the Montreal Cognitive Assessment, the informant questionnaire on cognitive decline, and a formal neuropsychological battery, all of which had been validated for older Singaporeans. The neuropsychological battery assessed seven cognitive domains, two of which were memory domains: verbal (word list recall and story recall) and visual (picture recall and Wechsler memory scale-revised visual reproduction) memories^52^ (see details in supplementary). Both visual and verbal memory domain scores were combined into a composite memory z-score for further analyses.

Both aMCI and AD diagnoses were made at weekly consensus meetings in which clinical features, blood tests, psychometrics, and neuroimaging data were reviewed^52^. Computed tomography (CT), magnetic resonance imaging (MRI), and magnetic resonance angiography were reviewed as part of the diagnostic process. Clinical AD was diagnosed according to the Diagnostic and Statistical Manual of Mental Disorders IV criteria (DMS-IV) and the National Institute of Neurological and Communicative Disorders and Stroke and the AD and Related Disorders Association guidelines for AD^52^. AD patients had a gradual and slow onset of memory problems, impairment in objective neuropsychological assessment, and loss of activities of daily living. All the AD patients had CDR global ≥1 and CDR sum of box ≥4. Clinical aMCI was diagnosed based on: (i) subjective complaints of memory loss, (ii) memory (verbal or visual) impairment on neuropsychological assessment described above, and (iii) absence of diagnosed dementia based on the DSM-IV criteria^53,54^. All the aMCI patients had CDR global <1 and CDR sum of box <4. We excluded participants with significant cerebrovascular disease or psychiatric/neurologic disorders^55^ (see details in Supplementary). For the healthy controls (HC), we ensured that the participants had no impairment in the seven domains, their MMSE scores were greater than or equal to 26, and CDR were equal to 0^56,57^.

Of the 172 eligible HC, aMCI and AD subjects who were selected between August 12, 2010, and June 22, 2016, 5 participants did not have full MRI scans; 16 participants did not pass quality control criteria for structural MRI, resting-state functional MRI, or DTI (see quality control criteria in supplementary); and 5 participants did not complete the neuropsychological assessments. The remaining 151 participants (51 HC, 54 aMCI, 46 AD) were included in the analyses (Table 1).

**Table 1:** Demographic and neuropsychological features of subjects.

*Groups*	HC (n = 51)	aMCI (n = 54)	AD (n = 46)	Overall ANOVA P value
Age	72.0 (4.1)	73.5 (7.9)	75.2 (7.9)	p = 0.08
Gender (F/M)	35/16	31/23	31/15	p = 0.42 (χ^2^)
Handedness (L/R)	3/48	3/51	1/45	p = 0.63 (χ^2^)
Ethnicity (C/N)	43/8	47/7	38/8	p = 0.82 (χ^2^)
Education	8.8 (4.6)	6.8 (5.1)	5.0 (4.7)^c^	**p** = **0.01**
MOCA (max = 30)	25.3 (2.7)	19.1 (4.6)^c^	11.5 (4.9)^mc^	**p** < **0.001**
CDR-global	0 (0)	0.4 (0.2)^c^	1.2 (0.4)^mc^	**p** < **0.001**
CDR-SB	0 (0)	0.8 (0.8)^c^	6.7 (2.8)^mc^	**p** < **0.001**
MMSE (max = 30)	28.2 (1.8)	23.7 (4.1)^c^	16.2 (5.3)^mc^	**p** < **0.001**
Visual construction (max = 32)	20.6 (4.3)	16.4 (4.2)^c^	10.1 (5.3)^mc^	**p** < **0.001**
Visual motor (max = 100)	34.8 (14.1)	39.7 (24.4)	54.5 (28.2)^mc^	**p** < **0.001**
Attention (max = 12)	8.4 (1.1)	6.9 (1.3)^c^	4.7 (2.2)^mc^	**p** < **0.001**
Executive functioning (max = 20)	16.9 (1.6)	14.2 (2.6)^c^	10.2 (3.7)^mc^	**p** < **0.001**
Language (max = 20)	16.8 (2.0)	13.0 (2.2)^c^	8.7 (3.2)^mc^	**p** < **0.001**
Verbal memory (max = 15)	9.8 (1.5)	4.8 (2.3)^c^	2.0 (1.3)^mc^	**p** < **0.001**
Visuospatial memory (max = 20)	11.2 (1.8)	6.9 (2.8)^c^	2.7 (1.8)^mc^	**p** < **0.001**

The values represent the means (SDs). Variables showing group differences (p < 0.05) are in bold. χ^2^ indicates that the χ^2^ test was used. Superscript letters indicate whether group mean was significantly worse than healthy control (HC) (c), amnestic mild cognitive impairment (aMCI) (m) based on post hoc pairwise comparisons (p < 0.05). Abbreviations: F/M: female/male; L/R: left/right; C/N: Chinese/non-Chinese; MOCA: Montreal Cognitive Assessment score, CDR-SB: Clinical Dementia Rating, sum-of-boxes; MMSE: Mini-Mental State Examination.

### Image acquisition

Each subject underwent MRI scanning at the Clinical Imaging Research Centre, National University of Singapore (3-T MAGNETOM Trio™, A Tim^®^ System; Siemens, Germany). High-resolution T1-weighted structural MRI was performed using a magnetization-prepared rapid gradient echo (MPRAGE) sequence (192 continuous sagittal slices, repetition time (TR) = 2300 ms, echo time (TE) = 1.9 ms, inversion time = 900 ms, flip angle = 9˚, field of view (FOV) = 256 × 256 mm^2^, matrix = 256 × 256, isotropic voxel size = 1-mm isotropic, bandwidth = 240 Hz/pixel). Diffusion MRI scans were acquired using a single-shot fast echo-planar imaging sequence (TR = 6800 ms, TE = 85 ms, slices = 48, FOV = 256 × 256 mm^2^, voxel size = 3-mm isotropic, b value = 1150 s/mm^2^, 61 diffusion directions, and 7 b0). A 5-minute task-free functional MRI scan was acquired using a T2*-weighted echo-planar sequence (TR= 2300 ms, TE = 25 ms, flip angle = 90˚, FOV = 192 × 192 mm^2^, voxel size = 3-mm isotropic, and 48 axial slices, with interleaved acquisition). Fluid-attenuated inversion recovery (FLAIR) imaging was also performed (TR = 11,000 ms, TE = 125 ms, inversion time = 2,800 ms, FOV = 256 × 256 mm^2^, sensitivity encoding factor 1.5, voxel size = 1.02 × 1.02 mm^2^, 60 slices, and slice thickness = 2.5 mm).

### Diffusion MRI data pre-processing

The diffusion MRI data were pre-processed using FSL (http://www.fmrib.ox.ac.uk/fsl)^32^. Head movements and eddy current distortions were corrected to the first b = 0 volume via affine registration of the diffusion-weighted images. Data were discarded if the maximum displacement relative to the first b = 0 volume was greater than 3 mm. The diffusion gradients were rotated to compensate for the registration. Individual maps were visually inspected for signal dropout, artefacts, and additional motion. Individual fractional anisotropy (FA) maps were created by fitting the DTI model to the pre-processed diffusion data at each voxel. FA images were non-linearly registered to the high-resolution (1 mm^3^) FMRIB58 FA image and then skeletonized using TBSS for further statistical analysis.

### Free-water imaging method

We employed the free-water imaging method on the pre-processed diffusion MRI data to estimate the fractional volume of freely diffusing extracellular water molecules (FW) and the fractional anisotropy of water molecules in the proximity of tissue (FA_T_)^14,15^. Briefly, the FW compartment models water molecules that are free to diffuse and not restricted or hindered during the diffusion process. This compartment has a fixed diffusivity of 3 × 10^−3^ mm^2^/s (the diffusion coefficient of free-water at body temperature), and the fractional volume of this compartment in each voxel forms the FW map. The FW-corrected DTI compartment models water molecules in the proximity of cellular membranes of brain tissue using a diffusion tensor, from which the FA_T_ measure is derived. Therefore, the FW-corrected DTI compartment is corrected for contamination with freely diffusing extracellular water and is consequently expected to be more sensitive and specific to axonal changes than the measures derived from the single tensor model^33^. Voxel-wise FW and FA_T_ were obtained for each subject^16^. The aligned FW and FA_T_ maps of each participant were then projected onto the standardized FA skeleton, resulting in subject-level skeletonized images.

### Voxel-based morphometry

We applied optimized voxel-based morphometry (Computational Anatomy Toolbox 12) using Statistical Parametric Mapping (SPM12)^55^. Briefly, we derived the subject-level GMV probability maps from the T1 structural images using an approach that included: (1) segmentation of individual T1-weighted images into the GM, WM and CSF; (2) creation of a study-specific template using non-linear DARTEL (Diffeomorphic Anatomical Registration Through Exponentiated Lie Algebra) registration of the affine-registered GM and WM segments; (3) registration of each GM/WM probability map to the study-specific template in Montreal Neurological Institute (MNI) space; (4) modulation by multiplying the voxel values by the Jacobian determinants to account for individual brain volumes; and (5) smoothing of the normalized GM maps by a 8-mm isotropic Gaussian kernel.

### Functional image pre-processing

Task-free functional MRI images were pre-processed using the Analysis of Functional NeuroImages software (https://afni.nimh.nih.gov/) and FSL^55^. The pre-processing steps included: (1) removal of the first five volumes to allow for magnetic field stabilization; (2) motion correction; (3) time series de-spiking; (4) spatial smoothing; (5) grand mean scaling; (6) band pass temporal filtering; (7) removal of linear and quadratic trends; (8) co-registration of T1 images using boundary-based registration and subsequent registration of the functional images into an MNI-152 space using a non-linear registration tool (FNIRT); and (9) regression of nine nuisance signals (WM, CSF, global signals and six motion parameters) from the pre-processed functional images. To determine whether global signal regression was preferred, we calculated the global negative index for each subject, taken as the percentage of voxels showing a negative correlation with the global signal^55^. Majority of our subjects (90.1%) had the global negative index of <3%, suggesting that the global signal was more representative of non-neural noise and should be regressed out from the images.

### Functional connectivity analyses

Individual-level DMN functional connectivity maps were obtained using a seed-based approach with the REST toolbox^58^. We created spherical region of interest (ROIs) with a 4-mm radius centred at the left posterior cingulate cortex (MNI coordinates [−7, −43, 33]). This seed was previously determined as a core region of DMN^48,59^. Pearson’s correlations were then computed between the time-series of every voxel in the brain and the average time series of the seed ROI. The FC correlation maps were converted to z-score maps using Fisher’s r-to-z transformation.

### Statistical analyses

We analysed the demographic, clinical, and cognitive measures across groups via ANOVA or *χ*^2^ tests using Statistical Package for Social Sciences (SPSS v. 23.0) software. The results were reported at a significance level of p < 0.05.

#### Associations between brain structure/functional measures and memory impairment

At the first step, to identify region-specific WM changes underlying memory deficit in patients, we built voxel-wise general linear models (GLMs) with the skeletonized FW and FA_T_ images as the dependent variables separately using the FSL. In each model, the memory domain z-score was the independent variable of interest, with age, gender, handedness and ethnicity as covariates. Regions were examined for statistical significance using threshold-free cluster enhancement (TFCE) and permutation-based non-parametric testing (FSL Randomise). Results were family-wise error (FWE) corrected at p < 0.05.

To examine the association between GMV and memory function among the aMCI and AD patients, we built the voxel-wise GLMs using SPM12 toolbox, with a threshold at p < 0.05, FWE corrected. To examine whether and how FC within the DMN related to memory performance across the aMCI and AD patients, we built voxel-wise GLMs using the REST toolbox^58^. Analysis was restricted to the DMN based a predefined group-level mask derived from an independent group of healthy control subjects^55^. The results were reported at a height threshold of p < 0.01 and a cluster threshold of p < 0.05 with Gaussian random field (GRF) correction^58^. We then extracted the mean values of brain structural/functional measures from the resulting significant regions for further statistical analyses.

#### Sparse varying coefficient (SVC) modelling of severity-dependent associations between brain measures and memory impairment

In reality, the differential pathophysiologies in GM and WM might interact with each other to influence with memory in AD^18^. Furthermore, there are no firm boundaries between the various clinical stages^1^. Therefore, in the second step, we employed the SVC model^31,32^ to integrate all structural and functional measures derived from the previous screening step as predictors in the same model to evaluate their relative contribution to and severity-dependent (CDR sum-of-boxes (CDR-SB) as a measure of dementia severity) impact on memory, which provides a more comprehensive and nuanced picture. Specifically, we tested whether and how the associations of brain function/structures with memory were dependent on dementia severity using memory z-scores as the dependent variable:

$${y}_{i}({t}_{k})=\sum _{j=1}^{p}{\beta }_{j}({t}_{k}){x}_{ij}({t}_{k})+{\varepsilon }_{i}({t}_{k}),$$

where *y*_*i*_ (*t*_*k*_) represents the memory z-scores for subject *i*(*i* = 1, 2, …, *n*) at the dementia severity *t*_*k*_, measured by CDR-SB. *x*_*ij*_ (*t*_*k*_) is the *j*^th^ (*j* = 1, 2, …, *p*) predictor of subject *i* at CDR-SB *t*_*k*_. *β*_*j*_ (*t*_*k*_) is the estimated coefficient function depending on CDR-SB *t*_*k*_ for each predictor. *ε*_*i*_ (*t*_*k*_) represents the independent and identically distributed random errors at *t*_*k*_.

For predictors *x*_*ij*_ (*t*_*k*_), we extracted the mean values from the previous identified candidate regions of interest (i.e., FW, FA_T_, FC-DMN, and GMV from mPFC, PCC, hippocampus (HIP)). All predictors were put in the same model with age, gender, handedness, and ethnicity included as nuisance variables. Each predictor was standardized to have zero mean and equal variance across observations. To simultaneously achieve regression model fitting and predictor variable selection, we applied the least absolute shrinkage and selection operator (LASSO)^60^ to estimate *β*_*j*_ (*t*_*k*_) by minimizing the following penalized least squares function.

$$\frac{1}{2n}\sum _{i=1}^{n}\sum _{k=1}^{K}{[{y}_{i}({t}_{k})-\sum _{j=1}^{p}{x}_{ij}({t}_{k}){\beta }_{j}({t}_{k})]}^{2}+\lambda \sum _{j=1}^{p}\sqrt{{\int }^{}{\beta }_{j}^{2}(t)dt},$$

where *λ* is the sparsity penalty tuning parameter chosen by a five-fold cross-validation method. The LASSO algorithm performs variable selection by constraining the sum of the squared magnitudes of the coefficients. SVC modelling with the LASSO algorithm was specifically designed for feature selection problems with small sample sizes^31^. We approximated each coefficient function *β*_*j*_ using linear combinations of the B-spline basis (number of basis functions *L* = 4).

Our SVC model offers several advantages over a traditional linear regression model: (i) it does not assume that the association of the brain measures with memory remains constant over disease progression and thus considers each beta coefficient (the association of brain function or structure with memory) as a non-linear function of a continuous variable of dementia severity (i.e., CDR-SB); (ii) feature selection with the LASSO sparsity penalty chooses the most important predictors while eliminating the contributions of the less important predictors; and (iii) rather than analysing brain measures in separate models, all variables are entered as predictors in the same multivariate model.

To assess the stability of these beta coefficients, we calculated the means and standard errors of the severity-dependent coefficients estimated from 100 replicates. We reported the brain measures that were selected in all 100 repetitions of SVC modelling. SVC modelling was performed by in-house R scripts based on Daye and colleagues^31^.

## Supplementary information

Supplemental information

## Data Availability

The data that support the findings of this study are available from Memory Ageing and Cognition Centre (MACC) but restrictions apply to the availability of these data, which were used under license for the current study, and so are not publicly available. Data are however available from the authors upon reasonable request and with permission of MACC.

## References

[1] Aisen PS (2017). On the path to 2025: understanding the Alzheimer’s disease continuum. Alzheimers Res Ther.

[2] Zhuang L (2012). Microstructural white matter changes in cognitively normal individuals at risk of amnestic MCI. Neurology.

[3] Petersen RC (2004). Mild cognitive impairment as a diagnostic entity. J Intern Med.

[4] Sperling, R. The potential of functional MRI as a biomarker in early Alzheimer’s disease. *Neurobiology of aging* (2011). 10.1016/j.neurobiolaging.2011.09.009 PMC3233699 22078171

[5] Jones DT (2016). Cascading network failure across the Alzheimer’s disease spectrum. Brain.

[6] Teipel S (2016). Measuring Cortical Connectivity in Alzheimer’s Disease as a Brain Neural Network Pathology: Toward Clinical Applications. J Int Neuropsychol Soc.

[7] Pengas G, Hodges JR, Watson P, Nestor PJ (2010). Focal posterior cingulate atrophy in incipient Alzheimer’s disease. Neurobiol Aging.

[8] Buckner RL, Andrews-Hanna JR, Schacter DL (2008). The brain’s default network: anatomy, function, and relevance to disease. Ann N Y Acad Sci.

[9] Eichenbaum H (2017). Prefrontal-hippocampal interactions in episodic memory. Nat Rev Neurosci.

[10] He J (2012). Influence of functional connectivity and structural MRI measures on episodic memory. Neurobiol Aging.

[11] Sachdev PS, Zhuang L, Braidy N, Wen W (2013). Is Alzheimer’s a disease of the white matter?. Curr Opin Psychiatry.

[12] Mielke MM (2012). Fornix integrity and hippocampal volume predict memory decline and progression to Alzheimer’s disease. Alzheimers Dement.

[13] Kantarci K (2014). Fractional anisotropy of the fornix and hippocampal atrophy in Alzheimer’s disease. Front Aging Neurosci.

[14] Pasternak O, Sochen N, Gur Y, Intrator N, Assaf Y (2009). Free water elimination and mapping from diffusion MRI. Magn Reson Med.

[15] Ji F (2017). Distinct white matter microstructural abnormalities and extracellular water increases relate to cognitive impairment in Alzheimer’s disease with and without cerebrovascular disease. Alzheimers Res Ther.

[16] Pasternak O, Westin CF, Dahlben B, Bouix S, Kubicki M (2015). The extent of diffusion MRI markers of neuroinflammation and white matter deterioration in chronic schizophrenia. Schizophr Res.

[17] Scheltens P (2016). Alzheimer’s disease. The Lancet.

[18] Jack CR, Holtzman DM (2013). Biomarker modeling of Alzheimer’s disease. Neuron.

[19] Sheline YI, Raichle ME (2013). Resting state functional connectivity in preclinical Alzheimer’s disease. Biol Psychiatry.

[20] Jack CR (2013). Tracking pathophysiological processes in Alzheimer’s disease: an updated hypothetical model of dynamic biomarkers. Lancet Neurol.

[21] Bateman RJ (2012). Clinical and biomarker changes in dominantly inherited Alzheimer’s disease. N Engl J Med.

[22] McDonald CR (2009). Regional rates of neocortical atrophy from normal aging to early Alzheimer disease. Neurology.

[23] Mizuno K, Wakai M, Takeda A, Sobue G (2000). Medial temporal atrophy and memory impairment in early stage of Alzheimer’s disease: an MRI volumetric and memory assessment study. Journal of the neurological sciences.

[24] Fotenos AF, Snyder AZ, Girton LE, Morris JC, Buckner RL (2005). Normative estimates of cross-sectional and longitudinal brain volume decline in aging and AD. Neurology.

[25] Frisoni GB, Prestia A, Rasser PE, Bonetti M, Thompson PM (2009). *In vivo* mapping of incremental cortical atrophy from incipient to overt Alzheimer’s disease. J Neurol.

[26] Wang L (2003). Changes in hippocampal volume and shape across time distinguish dementia of the Alzheimer type from healthy aging. Neuroimage.

[27] Fan Z, Brooks DJ, Okello A, Edison P (2017). An early and late peak in microglial activation in Alzheimer’s disease trajectory. Brain.

[28] Iturria-Medina Y (2016). Early role of vascular dysregulation on late-onset Alzheimer’s disease based on multifactorial data-driven analysis. Nat Commun.

[29] Moodley KK, Chan D (2014). The hippocampus in neurodegenerative disease. Front Neurol Neurosci.

[30] Boyle PA (2017). Varied effects of age-related neuropathologies on the trajectory of late life cognitive decline. Brain.

[31] Daye ZJ, Xie J, Li H (2012). A Sparse Structured Shrinkage Estimator for Nonparametric Varying-Coefficient Model with an Application in Genomics. J Comput Graph Stat.

[32] Hong Z (2015). Differential age-dependent associations of gray matter volume and white matter integrity with processing speed in healthy older adults. Neuroimage.

[33] Maier-Hein KH (2015). Widespread white matter degeneration preceding the onset of dementia. Alzheimers Dement.

[34] Rydhog AS (2017). Separating blood and water: Perfusion and free water elimination from diffusion MRI in the human brain. Neuroimage.

[35] Van de Haar HJ (2016). Blood-Brain Barrier Leakage in Patients with Early Alzheimer Disease. Radiology.

[36] Lenart N, Brough D, Denes A (2016). Inflammasomes link vascular disease with neuroinflammation and brain disorders. J Cereb Blood Flow Metab.

[37] Oishi K, Lyketsos CG (2014). Alzheimer’s disease and the fornix. Front Aging Neurosci.

[38] Badea A (2016). The fornix provides multiple biomarkers to characterize circuit disruption in a mouse model of Alzheimer’s disease. Neuroimage.

[39] Metzler-Baddeley C, Jones DK, Belaroussi B, Aggleton JP, O’Sullivan MJ (2011). Frontotemporal connections in episodic memory and aging: a diffusion MRI tractography study. J Neurosci.

[40] Villain N (2008). Relationships between hippocampal atrophy, white matter disruption, and gray matter hypometabolism in Alzheimer’s disease. J Neurosci.

[41] Mattsson N (2015). Brain structure and function as mediators of the effects of amyloid on memory. Neurology.

[42] Bejanin A (2017). Tau pathology and neurodegeneration contribute to cognitive impairment in Alzheimer’s disease. Brain.

[43] Palmqvist S (2017). Earliest accumulation of beta-amyloid occurs within the default-mode network and concurrently affects brain connectivity. Nat Commun.

[44] Nielsen FA, Balslev D, Hansen LK (2005). Mining the posterior cingulate: segregation between memory and pain components. Neuroimage.

[45] Zanto TP, Gazzaley A (2009). Neural suppression of irrelevant information underlies optimal working memory performance. J Neurosci.

[46] Dunn CJ (2014). Deficits in episodic memory retrieval reveal impaired default mode network connectivity in amnestic mild cognitive impairment. Neuroimage Clin.

[47] Fletcher E, Carmichael O, Pasternak O, Maier-Hein KH, DeCarli C (2014). Early Brain Loss in Circuits Affected by Alzheimer’s Disease is Predicted by Fornix Microstructure but may be Independent of Gray Matter. Front Aging Neurosci.

[48] Zhou J (2010). Divergent network connectivity changes in behavioural variant frontotemporal dementia and Alzheimer’s disease. Brain.

[49] Power JD, Plitt M, Laumann TO, Martin A (2017). Sources and implications of whole-brain fMRI signals in humans. Neuroimage.

[50] Caballero-Gaudes C, Reynolds RC (2017). Methods for cleaning the BOLD fMRI signal. Neuroimage.

[51] Glover GH, Li TQ, Ress D (2000). Image-based method for retrospective correction of physiological motion effects in fMRI: RETROICOR. Magn Reson Med.

[52] van Veluw SJ (2015). Cortical microinfarcts on 3T MRI: Clinical correlates in memory-clinic patients. Alzheimers Dement.

[53] Hilal S (2013). Prevalence of cognitive impairment in Chinese: epidemiology of dementia in Singapore study. J Neurol Neurosurg Psychiatry.

[54] Qiu Y (2016). Inter-hemispheric functional dysconnectivity mediates the association of corpus callosum degeneration with memory impairment in AD and amnestic MCI. Sci Rep.

[55] Chong JSX (2017). Influence of cerebrovascular disease on brain networks in prodromal and clinical Alzheimer’s disease. Brain.

[56] Hilal, S. *et al*. Prevalence of cognitive impairment and dementia in Malays - Epidemiology of Dementia in Singapore Study. *Curr Alzheimer Res* (2015). 10.2174/1567205012666151002123813 26428410

[57] Ong YT (2015). Retinal neurodegeneration on optical coherence tomography and cerebral atrophy. Neurosci Lett.

[58] Song XW (2011). REST: a toolkit for resting-state functional magnetic resonance imaging data processing. PloS one.

[59] Greicius MD, Srivastava G, Reiss AL, Menon V (2004). Default-mode network activity distinguishes Alzheimer’s disease from healthy aging: evidence from functional MRI. Proc Natl Acad Sci USA.

[60] Tibshirani, R. Regression shrinkage and selection via the lasso. *Journal of the Royal Statistical Society*. *Series B (Methodological)*, 267–288 (1996).

