# Supplementary material for: White matter microstructural abnormalities and default network degeneration are associated with early memory deficit in Alzheimer’s disease continuum
Source: Sci Rep. 2019 Mar 18;9:4749. doi: 10.1038/s41598-019-41363-2 (PMC6426923; doi:10.1038/s41598-019-41363-2)
Supplement: Supplementary file 1 — Supplemental information [file 41598_2019_41363_MOESM1_ESM.docx]

**SUPPLEMENTAL DATA**

**White matter microstructural abnormalities and default network degeneration are associated with early memory deficit in Alzheimer’s disease continuum**

Fang Ji, PhD^1^; Ofer Pasternak, PhD^2^; Kwun Kei Ng, PhD ^1^; Joanna Su Xian Chong, BS^1^; Siwei Liu, PhD^1^; Liwen Zhang, PhD^1^; Hee Youn Shim, BE^1^; Yng Miin Loke, BS^1^; Boon Yeow Tan, FCFP^3^; Narayanaswamy Venketasubramanian, FRCP^4^; Christopher Li-Hsian Chen, MD^5,6^; and Juan Helen Zhou, PhD^1,7^

^1^Center for Cognitive Neuroscience, Neuroscience and Behavioural Disorders Program, Duke-National University of Singapore Medical School, Singapore, ^2^Departments of Psychiatry and Radiology, Brigham and Women's Hospital, Harvard Medical School, USA, ^3^St Luke's Hospital, Singapore, ^4^Raffles Neuroscience Centre, Raffles Hospital, Singapore, ^5^Department of Pharmacology, National University Health System, Clinical Research Centre, ^6^Memory, Aging & Cognition Centre, National University Health System, National University of Singapore, Singapore, ^7^Clinical Imaging Research Centre, the Agency for Science, Technology and Research, Singapore

**Supplementary Methods**

**1. Neuropsychological evaluation, diagnoses and inclusion/exclusion criteria**

Each participant underwent extensive clinical and neuropsychological evaluation including the Clinical Dementia Rating Scale (CDR), the Mini-Mental State Examination (MMSE), the Montreal Cognitive Assessment, the informant questionnaire on cognitive decline and a formal neuropsychological battery, which had been locally validated for older Singaporeans. This battery assesses seven domains, five of which are non-memory domains. The non-memory domains of battery included: (a) executive function (frontal assessment battery^1^ and maze task^2^); (b) attention (digit span, visual memory span^3^ and auditory detection tests^4^); (c) language (Boston naming test ^5^ and verbal fluency ^6^); (d) visuomotor speed (symbol digit modality test^7^ and digit cancellation^8^); (e) visuoconstruction (Weschler memory scale revised visual reproduction copy task^3^, clock drawing^9^ and Weschler adult intelligence scale-revised subtest of block design^10^. The memory domains included: (a) verbal memory (word list recall ^11^ and story recall); (b) visual memory (picture recall and Weschler memory scale-revised visual reproduction^3^). The assessment was administered according to the subject**’**s habitual language and was completed in approximately 1 h.

Diagnoses of dementia were made at weekly consensus meetings, following a review of clinical history, blood markers, neuropsychological assessments and neuroimaging data by neurologists, neuropsychologists, research nurses, and research assistants (see previous work^12^). Diagnoses were consistent with the Diagnostic and Statistical Manual of Mental Disorders (DSM)-IV criteria. Alzheimer’s disease (AD) diagnoses were made according to the National Institute of Neurological and Communicative Disorders and Stroke and the AD and Related Disorders Association guidelines for AD^13^. AD patients had gradual and slow onset of memory problems, impairment in objective neuropsychological assessment, and loss in activities of daily living. Amnestic mild cognitive impairment (aMCI) were diagnosed based on: (1) subjective complaints of memory loss, (2) memory (verbal or visual) impairment on neuropsychological assessment, and (3) absence of diagnosed dementia^14^.

Subjects were also assessed for neuroimaging evidence of significant cerebrovascular disease. To minimize the potential confounding influence of cerebrovascular disease, subjects with the presence of any of the following: (1) cortical infarcts; (2) two or more lacunes; and (3) confluent white matter (WM) lesions in two brain regions (Age Related WM Change scale score ≥ 8)^15^ were excluded from the analysis^16^. Participants were further excluded from this study if they were/had: (1) hypoxic/anoxic, hypotensive, hypertensive, uremic or hepatic encephalopathy; (2) traumatic, nutritional or toxic disorders that affect the central nervous system (CNS); (3) current substance use disorders (e.g., alcohol, barbiturates, opiates, amphetamines, phencyclidine, and/or cocaine as defined by the DSM-IV) or previous substance use disorders that affect the CNS; (4) intracerebral hemorrhages that may cause cognitive impairments; (5) cranial arteritis, CNS inflammatory vasculitides, or Moyamoya disease; (6) CNS infections, including syphilis, tuberculosis, fungi, rickettsiae, bacterium, viral encephalitis, Creutzfeld-Jacob disease or other CNS infections; (7) space-occupying intracranial mass lesions; (8) obstructive or normal pressure hydrocephalus; (9) difficulty controlling epilepsy, which may cause cognitive impairments; or (10) schizophrenia or bipolar disorder.

**Supplementary Results**

**1. Group difference among HC, aMCI and AD**

We found aMCI had widespread white matter (WM) free-water (FW) increases and focal tissue FA_T_ damage in fornix, lateral frontal and parietal-occipital tracts compared with HC. aMCI had greater grey matter (GM) atrophy in the hippocampal/parahippocampal regions, precuneus, and lateral prefrontal cortex than HC. There was no significant difference in functional connectivity (FC) of the default mode network (DMN) between aMCI and HC (Supplementary Fig. 1).

AD had widespread FW increases and FA_T_ reduction compared with aMCI. Moreover, AD had greater GM atrophy in the hippocampal/parahippocampal regions, posterior cingulate cortex (PCC), and medial prefrontal cortex than aMCI. Lastly, we found AD patients had lower FC of the DMN in the precuneus and posterior cingulate cortex (PCC) regions than aMCI. (Supplementary Fig. 2).

Compared to the HC group, AD group had extensive WM FW increases, WM tissue FA_T_ reduction, GM atrophy, and DMN FC disruption in the PCC, precuneus and left angular gyrus/late temporal regions.

**2. Brain-memory association after controlling for years of education**

The whole-brain voxel-wise analysis on diffusion MRI metrics showed that lower memory scores in aMCI and AD patients were associated with higher FW in most WM regions. (Supplementary Fig. 5A). In contrast, lower memory score was associated with lower FA_T_ in the body of the fornix only (Supplementary Fig. 5B). Lower GMV in the bilateral MTL, PCC, and mPFC were associated with lower memory scores across all patients (Fig. 5C). Finally, lower memory score was associated with lower FC in the precuneus and angular gyrus across all patients (Supplementary Fig. 5D).

For the SVC model, FW (widespread), FA_T_ in the fornix, GMV-mPFC, GMV-PCC, GMV-HIP, and FC-DMN derived from the significant regions of voxel-wise analysis as predictors. We found these brain measures exhibited differential severity-dependent associations with memory (Supplementary Fig. 6). FW had the greatest influence on memory deficit in the early aMCI phase where higher FW was associated with lower memory score. However, this influence gradually decreased in late aMCI and AD stage. Similarly, the association of FA_T_ in the fornix with memory score was the greatest in early aMCI stage, where higher FA_T_ was associated with better memory score. However, this association quickly diminished in the AD stage. Both PCC and mPFC had the strongest associations with memory in the early aMCI stage, where larger volume was associated with better memory score. Similar to FA_T_, this relationship gradually diminished in the AD stage. In contrast, the relationship between hippocampus (and MTL) and memory were more evident in the late aMCI stage and peaked at the early AD phase where larger volume was associated with better memory. The association between FC-DMN and memory was evident throughout the disease continuum. Higher FC was associated with higher memory score regardless of severity.

**REFERENCE:**

1 Dubois, B., Slachevsky, A., Litvan, I. & B, B. P. The FAB: a frontal assessment battery at bedside. *Neurology* **55**, 1621-1626 (2000).

2 Porteus, S. D. *The Maze Test and Clinical Psychology*. (Pacific Books, 1959).

3 Wechsler, D. *Wechsler Memory Scale-revised*. Third edn, (The Psychological Corporation, 1997).

4 Lewis, R. & Rennick, P. *Manual for the Repeatable Cognitive Perceptual-Motor Battery*. (Axon, 1979).

5 Mack, W. J., Freed, D. M., Williams, B. W. & Henderson, V. W. Boston Naming Test: Shortened Versions for Use in Alzheimer's Disease. *Journal of Gerontology* **47**, P154-P158, doi:10.1093/geronj/47.3.P154 (1992).

6 ISAACS, B. & KENNIE, A. T. The Set Test as an Aid to the Detection of Dementia in Old People. *The British Journal of Psychiatry* **123**, 467-470, doi:10.1192/bjp.123.4.467 (1973).

7 Smith, A. *Symbol Digit Modalities Test*. (The American Psychiatric Pub, 1973).

8 Diller, L., Ben-Yishay, Y. & Gerstman, L. J. *Studies in cognition and rehabilitation in hemiplegia*. (Institute of Rehabilitation Medicine, New York University Medical Center 1974).

9 Sunderland, T., Hill, J. L. & Mellow, A. M. Clock drawing in Alzheimer’s disease. A novel measure of dementia severity. *American Geriatrics Society* **7**, 725-729 (1989).

10 Wechsler, D. *Wechsler Adult Intelligence Scale-Revised*. (Harcourt Brace Jovanovich, 1981).

11 Sahdevan, S., Tan, N. J. L., Tan, T. & Tan, S. Cognitive testing of elderly Chinese people in Singapore: influence of education and age on normative scores. *Age and Ageing* **26**, 481-486, doi:10.1093/ageing/26.6.481 (1997).

12 Narasimhalu, K. *et al.* Severity of CIND and MCI predict incidence of dementia in an ischemic stroke cohort. *Neurology* **73**, 1866-1872, doi:10.1212/WNL.0b013e3181c3fcb7 (2009).

13 Dubois, B. *et al.* Research criteria for the diagnosis of Alzheimer's disease: revising the NINCDS-ADRDA criteria. *Lancet Neurol* **6**, 734-746, doi:10.1016/S1474-4422(07)70178-3 (2007).

14 Qiu, Y. *et al.* Inter-hemispheric functional dysconnectivity mediates the association of corpus callosum degeneration with memory impairment in AD and amnestic MCI. *Sci Rep* **6**, 32573, doi:10.1038/srep32573 (2016).

15 Hilal, S. *et al.* Markers of cardiac dysfunction in cognitive impairment and dementia. *Medicine* **94**, e297, doi:10.1097/MD.0000000000000297 (2015).

16 Liu, S. *et al.* The Association Between Retinal Neuronal Layer and Brain Structure is Disrupted in Patients with Cognitive Impairment and Alzheimer's Disease. *Journal of Alzheimer's disease : JAD* **54**, 585-595, doi:10.3233/JAD-160067 (2016).

**Supplementary Table 1. Regions where white matter (WM) measures were associated with memory.** The WM clusters in which the free-water (FW), tissue compartment fraction anisotrophy (FA_T_) with memory (TFCE and FWE corrected, p < 0.01, with a minimum cluster size of 10 voxels) are listed with the MNI coordinates (mm) and t-statistics of the peak voxel. **Abbreviations:** left hemisphere = L, right hemisphere = R, corticospinal tract = CST, cerebral peduncle R = CP, anterior limb of internal capsule = ALIC, posterior limb of internal capsule = PLIC, retrolenticular part of internal capsule = RLIC, anterior corona radiata = ACR, superior corona radiata = SCR, posterior corona radiata = PCR, posterior thalamic radiation (include optic radiation) = PTR, external capsule = EC, sagittal stratum (includes inferior longitudinal fasciculus and inferior fronto-occipital fasciculus) = SS, superior longitudinal fasciculus = SLF, superior fronto-occipital fasciculus (may be part of the anterior internal capsule) = SFO, uncinate fasciculus = UF, fornix (column and body of the fornix) = Fx, cingulum (cingulate gyrus) = CCG, cingulum (hippocampus) = CHIP, fornix (cres)/stria terminalis (cannot be resolved with the current resolution) = Fx/ST, genu of the corpus callosum = gCC, body of the corpus callosum = bCC, splenium of the corpus callosum = sCC, tapetum = TAP, middle cerebellar peduncle = MCP, pontine crossing tract (a part of the MCP) = PCT, medial lemniscus = ML, inferior cerebellar peduncle = ICP, superior cerebellar peduncle = SCP.

| **Brain region** | | **FW** | | | | **FA_T_** | | | |
| --- | --- | --- | --- | --- | --- | --- | --- | --- | --- |
|  |  | **X** | **Y** | **Z** | **t-stats** | **X** | **Y** | **Z** | **t-stats** |
| **Projection Fibers** | R CST | 8 | -25 | -27 | 4.1 | - | - | - | - |
|  | L CST | -5 | -25 | -35 | 3.8 | - | - | - | - |
|  | R CP | 7 | -21 | -19 | 3.3 | - | - | - | - |
|  | L CP | -9 | -13 | -15 | 3.2 | - | - | - | - |
|  | R ALIC | 11 | 7 | -1 | 4.3 | - | - | - | - |
|  | L ALIC | -13 | 4 | 7 | 3.4 | - | - | - | - |
|  | R PLIC | 24 | -11 | 13 | 3.3 | - | - | - | - |
|  | L PLIC | -23 | -13 | 14 | 3.7 | - | - | - | - |
|  | R RLIC | 31 | -34 | 13 | 3.6 | - | - | - | - |
|  | L RLIC | -29 | -34 | 14 | 3.5 | - | - | - | - |
|  | R ACR | 23 | 21 | 8 | 3.6 | - | - | - | - |
|  | L ACR | -22 | 26 | -3 | 3.7 | - | - | - | - |
|  | R SCR | 18 | -15 | 37 | 3.2 | - | - | - | - |
|  | L SCR | -18 | -17 | 36 | 3.2 | - | - | - | - |
|  | R PCR | 29 | -37 | 20 | 3.0 | - | - | - | - |
|  | L PCR | -29 | -40 | 19 | 4.2 | - | - | - | - |
|  | R PTR | 32 | -42 | 18 | 4.8 | - | - | - | - |
|  | L PTR | -26 | -63 | 13 | 4.2 | - | - | - | - |
|  | R EC | 29 | 9 | -12 | 3.2 | - | - | - | - |
|  | L EC | -31 | 3 | -11 | 3.1 | - | - | - | - |
| **Association Fibers** | R SS | 38 | -43 | -7 | 4.1 | - | - | - | - |
|  | L SS | -41 | -26 | -15 | 3.2 | - | - | - | - |
|  | R SLF | 35 | -19 | 34 | 4.1 | - | - | - | - |
|  | L SLF | -32 | -20 | 34 | 4.7 | - | - | - | - |
|  | R UF | 35 | 1 | -17 | 3.3 | - | - | - | - |
|  | L UF | -32 | 2 | -12 | 4.7 | - | - | - | - |
| **Limbic Fibers** | Fx | 0 | 1 | 16 | 3.9 | 0 | -2 | 15 | 4.9 |
|  | R CCG | 7 | -5 | 37 | 4.1 | - | - | - | - |
|  | L CCG | -9 | -48 | 24 | 3.3 | - | - | - | - |
|  | L CHIP | -19 | -40 | -4 | 3.2 | - | - | - | - |
|  | R Fx/ST | 23 | -34 | 5 | 4.6 | - | - | - | - |
|  | L Fx/ST | -30 | -22 | -8 | 4.3 | - | - | - | - |
| **Callosal Fibers** | gCC | 1 | 28 | 4 | 3.0 | - | - | - | - |
|  | bCC | 12 | 0 | 30 | 3.5 | - | - | - | - |
|  | sCC | -7 | -39 | 16 | 4.5 | - | - | - | - |
|  | R TAP | 28 | -49 | 18 | 3.6 | - | - | - | - |
|  | L TAP | -27 | -51 | 18 | 3.7 | - | - | - | - |
| **Brainstem** | MCP | 12 | -35 | -30 | 3.1 | - | - | - | - |
|  | PCT | 3 | -22 | -25 | 3.3 | - | - | - | - |
|  | R ML | 6 | -34 | -31 | 3.1 | - | - | - | - |
|  | L ML | -4 | -35 | -35 | 3.8 | - | - | - | - |
|  | R ICP | 11 | -40 | -33 | 3.6 | - | - | - | - |
|  | R SCP | 6 | -37 | -23 | 3.2 | - | - | - | - |
|  | L SCP | -5 | -37 | -23 | 3.8 | - | - | - | - |

**Supplementary Table 2. Regions where grey matter (GMV) measures had positive correlation with memory.** GM clusters where volume was positively correlated with memory scores (FWE corrected, p < 0.05). Coordinates (x, y and z) are given in mm according to MNI space. **Abbreviations:** Hippocampus = HIP, Parahippocampus = PHIP, temporal pole: middle temporal gyrus = TPOmid, temporal pole: superior temporal gyrus = TPOsup, Amygdala = AMYG, Lingual gyrus = LING, Fusiform gyrus = FFG, middle temporal gyrus = MTG, Posterior cingulate gyrus = PCG, median cingulate and paracingulate gyri = DCG, Precuneus = PCUN, rectus gyrus = REC, superior frontal gyrus (medial) = ORBsup, superior frontal gyrus (medial orbital) = ORBsupmed.

| **Brain regions** | **MNI Coordinates** | | | | **Peak t Value** | **Cluster Size (Voxels)** |
| --- | --- | --- | --- | --- | --- | --- |
|  | **X** | **Y** | **Z** |  | |  |
| Right HIP/PHIP/TPOmid/MTG/FFG/ITG/AMYG/LING | 24 | -30 | -6 | 10.65 | | 12977 |
| Left HIP/PHIP/TPOmid/TPOsup/MTG/FFG/ITG/ LING | -22 | -32 | -4 | 12.31 | | 17549 |
| PCG/DCG/PCUN | 0 | -39 | 28 | 6.21 | | 1092 |
| REC/ORBsup/ORBsupmed | 12 | 44 | -18 | 6.24 | | 630 |

**Supplementary Table 3. Regions where default mode network functional connectivity had positive correlation with memory.** functional connectivity clusters where volume was positively correlated with memory score (height threshold of p < 0.01 and cluster threshold of p < 0.05, with Gaussian random field (GRF) correction). Coordinates (x, y and z) are given in mm according to MNI space. **Abbreviations:** Cuneus = CUN, Posterior cingulate gyrus = PCG, and Precuneus = PCUN.

| **Brain regions** | **MNI Coordinates** | | | **Peak t Value** | **Cluster Size (Voxels)** |  |
| --- | --- | --- | --- | --- | --- | --- |
|  | **X** | **Y** | **Z** |  |  | |
| PCUN/CUN/PCG | 10 | -72 | -32 | 4.83 | 958 | |

**Supplementary Figure 1. Brain structural and functional abnormalities in amnestic MCI patients than healthy controls.** (A) aMCI group had widespread free-water increase compared with HC (p < 0.05, threshold-free cluster enhancement and family-wise error corrected). The WM skeleton is highlighted in green. (B) aMCI group had tissue compartment fractional anisotropy (FA_T_) reduction in fornix, lateral frontal, and parietal-occipital tracts compared with HC (p < 0.05, threshold-free cluster enhancement and family-wise error corrected). (C) aMCI had greater grey matter atrophy in the hippocampal/parahippocampal (HIP) regions, precuneus (PreCu), and inferior frontal cortex than HC (p < 0.05, family-wise error corrected). (D) There was no significant difference in functional connectivity of the DMN between aMCI and HC (height threshold of p < 0.01 and a cluster threshold of p < 0.05, gaussian random field-corrected).

**
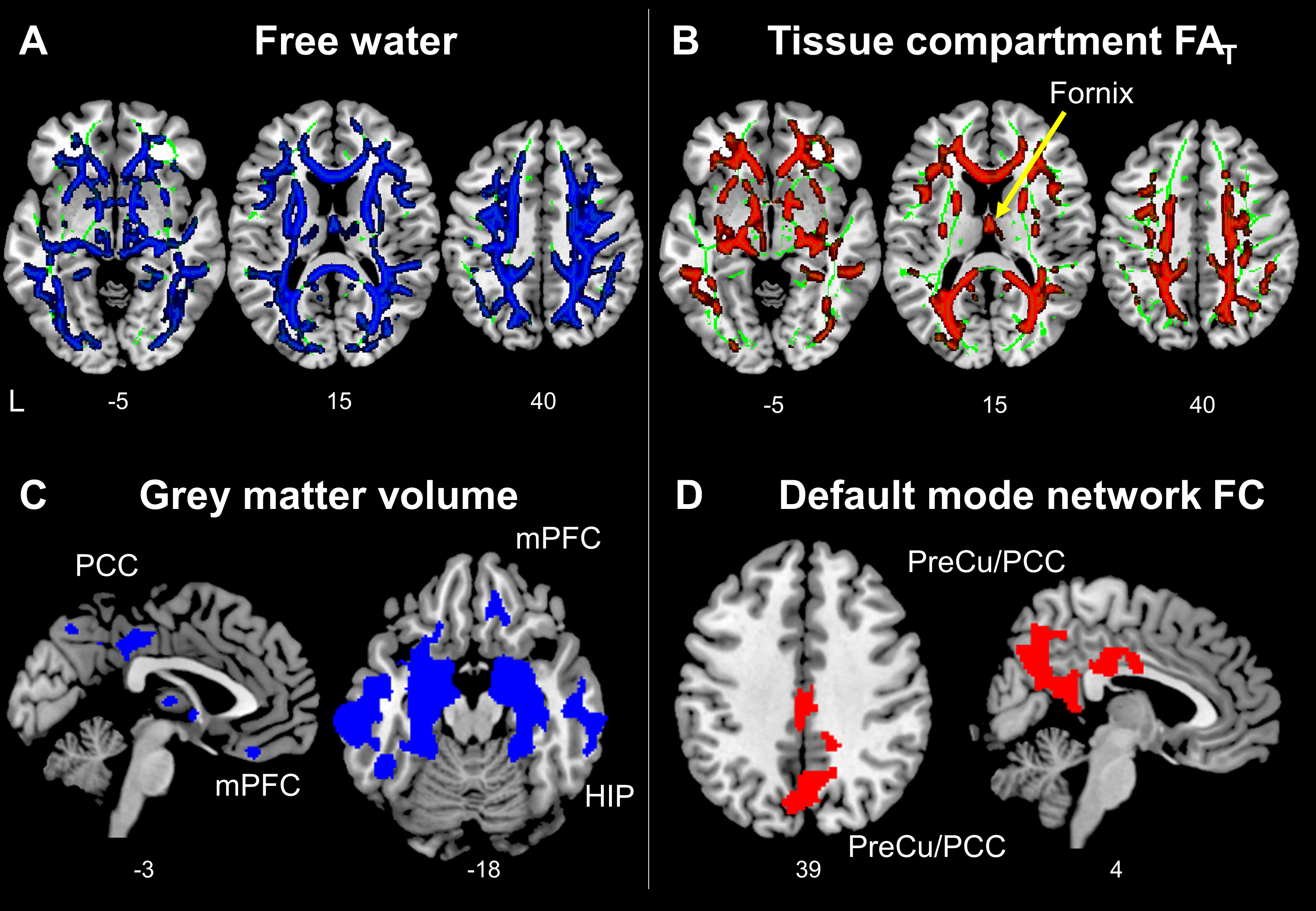
**

**Supplementary Figure 2. AD patients had greater brain abnormalities than aMCI.** (A) AD had widespread free-water increase compared with aMCI (p < 0.05, threshold-free cluster enhancement- and family-wise error-corrected). The WM skeleton is highlighted in green. (B) AD patients had widespread tissue compartment fractional anisotropy (FA_T_) reduction compared with aMCI (p < 0.05, threshold-free cluster enhancement- and family-wise error-corrected). (C) AD had greater grey matter atrophy in the hippocampal/parahippocampal (HIP) regions, posterior cingulate cortex (PCC), and medial prefrontal cortex (mPFC) than aMCI (p < 0.05, family-wise error-corrected). (D) AD patients had lower FC of the DMN in the precuneus (PreCu)/PCC regions than aMCI (height threshold of p < 0.01 and a cluster threshold of p < 0.05, gaussian random field-corrected).

**
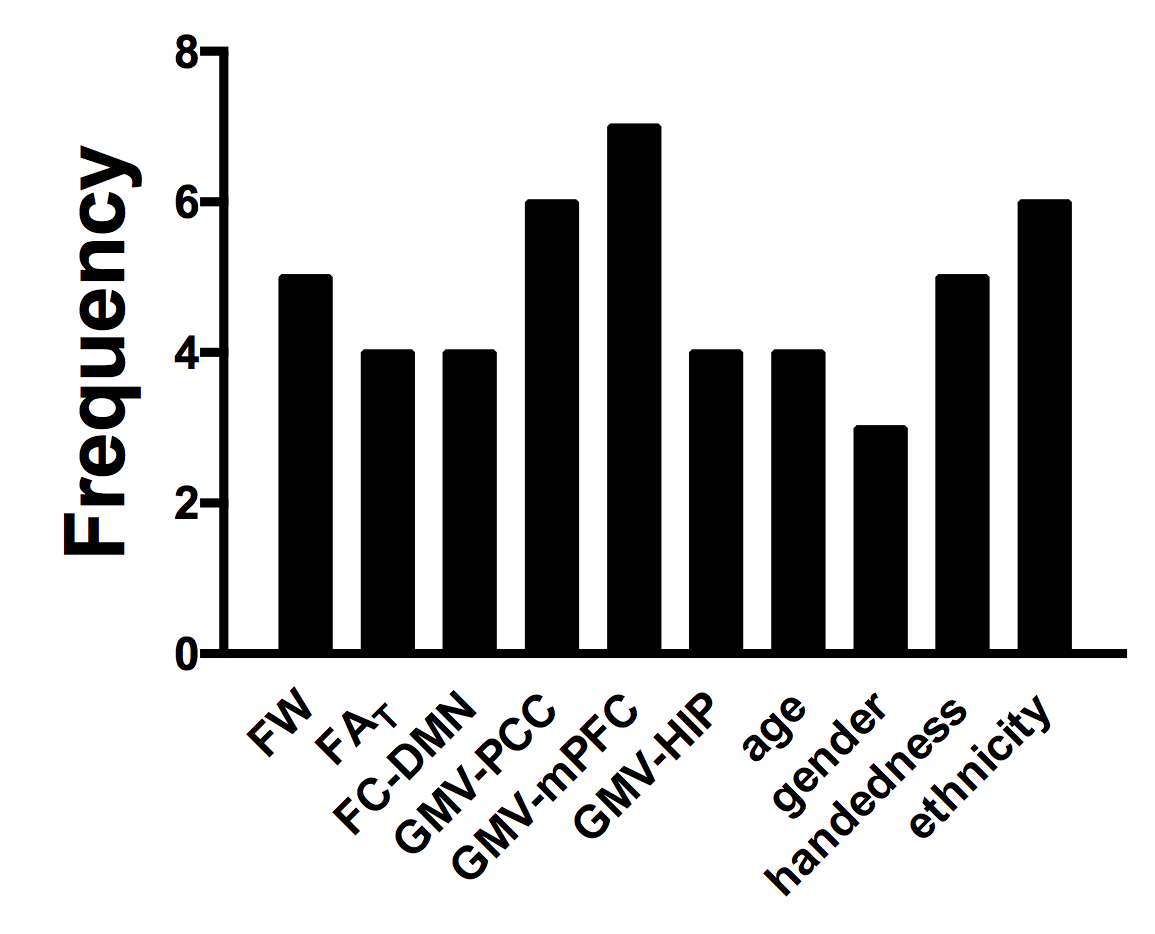
**

**Supplementary Figure 3. Variable selection frequency distribution for permuted datasets using sparse varying-coefficient (SVC) model.** To assess the false identification rate of identifying brain measures associated with memory scores in a severity-dependent manner, we applied the SVC model on 100 permuted data sets. The frequency distribution of each variable being selected by SVC models across these datasets was approximately random. The brain measurements (highlighted in text) identified by SVC model based on the original data was not favoured. For the rest permuted data sets, no variable was selected by all 100 repetitions.

**
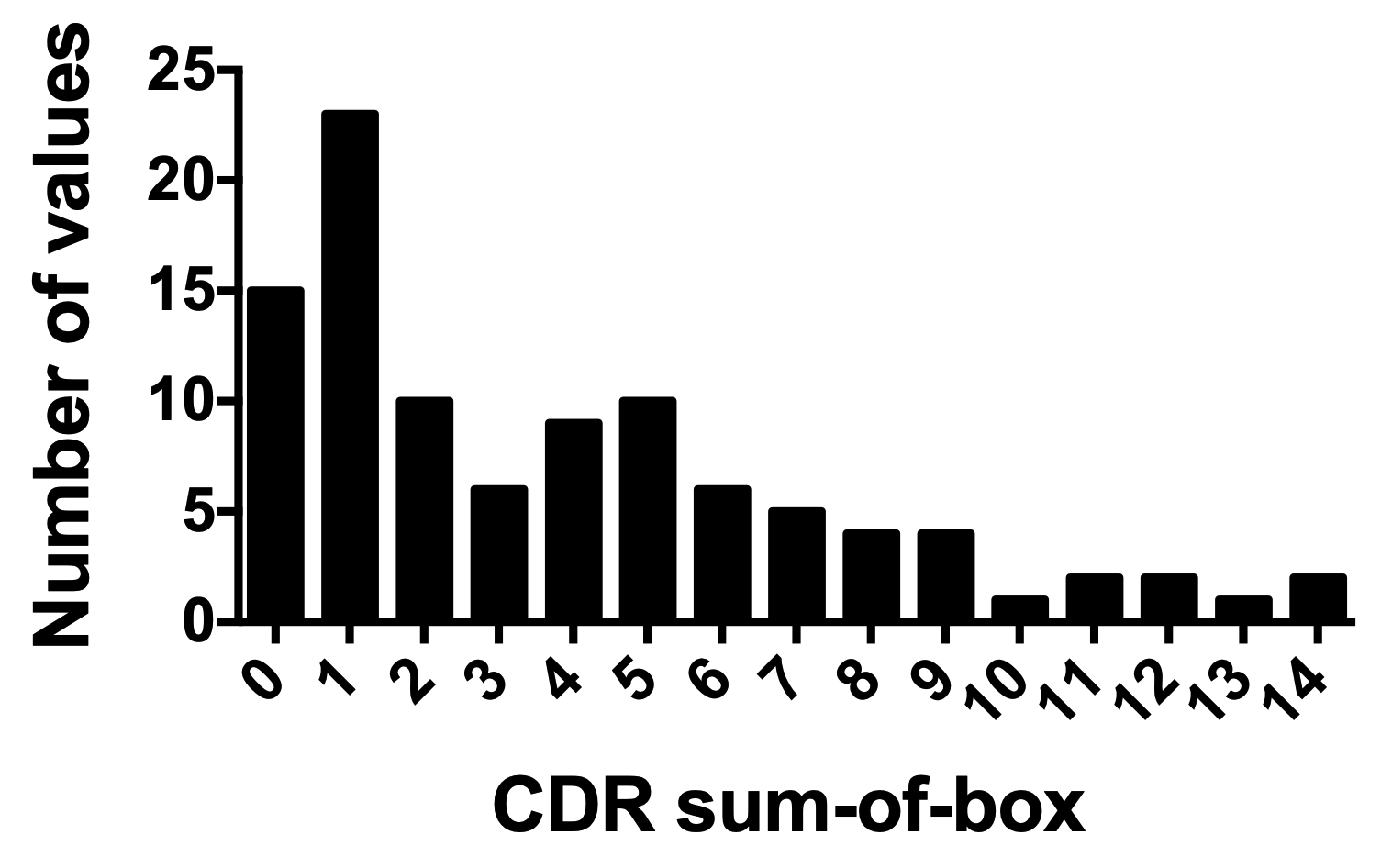
**

**Supplementary Figure 4. Frequency distribution of Clinical Dementia Rating Scale (CDR) sum-of-box in sparse varying-coefficient model.**

**Supplementary Figure 5. Free-water (FW) increases, tissue compartment fractional anisotropy (FA_T_) deterioration, grey matter volume (GMV) loss and default mode network (DMN) functional connectivity (FC) disruption correlated with verbal memory deficit in controlling for education.** (A) Whole-brain voxel-wise linear regression analysis indicated that higher FW values in widespread brain regions were associated with poorer memory. (B) Lower FA_T_ in the body of the fornix was associated with worse memory. The WM skeleton is highlighted in green. All the results are threshold-free cluster enhancement and family-wise error-corrected at p < 0.05. (C) Whole-brain voxel-wise linear regression analysis indicated that more grey matter atrophy in the hippocampal/parahippocampal (HIP) regions, posterior cingulate cortex (PCC), and medial prefrontal cortex (mPFC) was associated with worse memory (p < 0.05, family-wise error-corrected). (D) Lower FC of the DMN in the precuneus (PreCu) and angular gyrus (ANG) regions was associated with worse memory (height threshold of p < 0.01 and a cluster threshold of p < 0.05, gaussian random field-corrected).


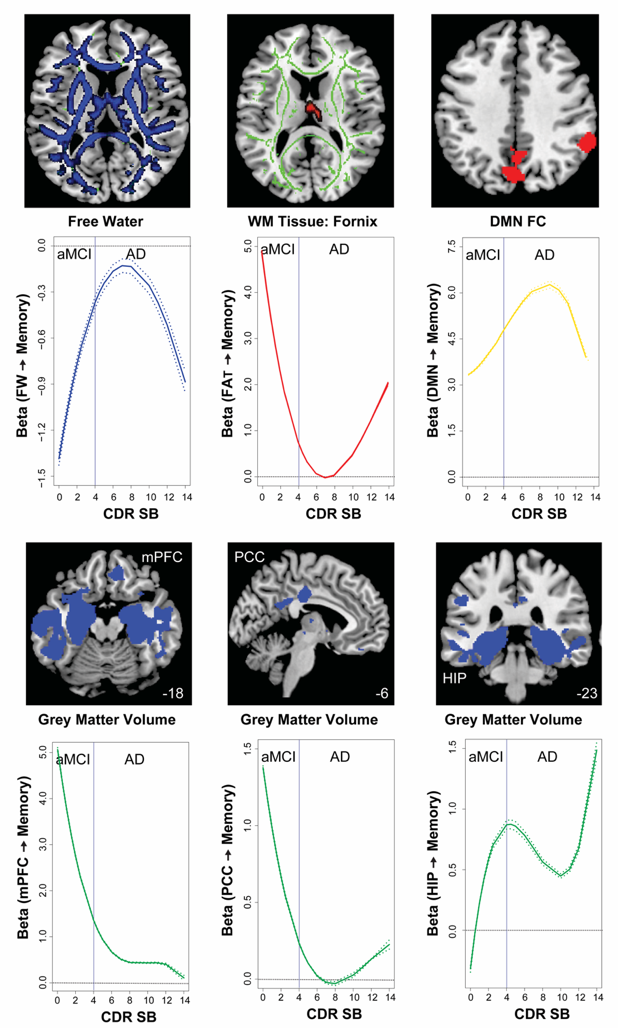


**Supplementary Figure 6. Severity-dependent associations of free water (FW), tissue compartment fractional anisotropy (FA_T_), grey matter volume (GMV), and functional connectivity (FC) with memory performance derived from a sparse varying coefficient model controlling for education.** Solid curves represent the mean associations (Beta coefficients) of brain measurements, with memory as a function of dementia severity (represented by the Clinical Dementia Rating Scale, sum-of-boxes (CDR-SB)) estimated from 100 replicates. The dashed curves represent the point-wise 2* standard errors of the solid curves estimated from 100 replicates. The horizontal dashed black lines represent Beta = 0. Abbreviations: HIP: hippocampus, PCC: posterior cingulate cortex, mPFC: medial prefrontal cortex.
